# Clinical outcomes of unilateral versus bilateral percutaneous vertebroplasty under local anaesthesia: a prospective randomised study

**DOI:** 10.3389/fsurg.2026.1744425

**Published:** 2026-03-27

**Authors:** Igor Movrin

**Affiliations:** Department of Traumatology, University Medical Centre Maribor, Maribor, Slovenia

**Keywords:** bilateral percutaneous vertebroplasty, local anaesthesia, osteoporosis, osteoporotic vertebral compression fracture, unilateral percutaneous vertebroplasty

## Abstract

Percutaneous vertebroplasty (PVP) is widely used to treat painful osteoporotic vertebral compression fractures (OVCFs), yet the comparative value of unilateral vs. bilateral access remains debated. The aim of this study was to compare unilateral and bilateral percutaneous vertebroplasty performed under local anaesthesia in patients with single-level OVCFs. This randomised study was conducted on 196 adults (mean age 71.7 ± 6.5 years) with single-level OVCFs treated under local anaesthesia and followed at baseline, discharge, and at the 6-month follow-up. Patients were allocated to unilateral or bilateral PVP. The primary outcomes were pain assessed on the visual analogue scale (VAS) and disability evaluated with the Oswestry Disability Index (ODI). Secondary endpoints included operative and fluoroscopy time, cement volume, vertebral body height restoration, kyphotic angle correction, and complications. Both techniques achieved rapid and significant pain relief, evident at discharge and sustained through the 6-month follow-up. Similarly, ODI demonstrated parallel improvement in both groups. Mean operative and fluoroscopy time was significantly shorter for unilateral PVP (27.4 ± 2.4 min) than with bilateral PVP (42.4 ± 3.0 min, *p* < 0.001), and fluoroscopy time was reduced by nearly 40% (58.2 ± 9.0 s vs. 93.8 ± 8.5 s, *p* < 0.001). No significant differences were found in vertebral height restoration and kyphotic angle correction. Cement volume was significantly lower with unilateral PVP (3.9 ± 0.4 mL) compared to bilateral PVP (5.8 ± 0.5 mL, *p* < 0.001)**.** Complication rates were low and comparable, with cement leakage observed in 7.1% of unilateral and 8.2% of bilateral cases, and new adjacent fractures in 5.1% vs. 8.2% respectively. Unilateral PVP confers some procedural advantages (time, radiation, cement use) without compromising clinical or radiographic outcomes at 6 months.

## 1 Introduction

Osteoporotic vertebral compression fractures (OVCF) are among the most common fractures in elderly patients. They can lead to significant pain and functional limitations, alongside a sizable impact on patients' quality of life and independence in activities of daily living (1, 2). Because of the ageing population and severe morbidity associated with these fractures, painful OVCFs are a growing, serious public health problem with critical socioeconomic effects (3).

With the development of minimally invasive concepts, percutaneous vertebroplasty (PVP) has become an essential method for the treatment of OVCFs. In PVP, bone cement (polymethylmethacrylate) is injected percutaneously into the fractured vertebral body under imaging guidance. The benefits of PVP include reduced tissue trauma, a shorter surgery time and an accelerated recovery period postoperatively (4).

Despite its widespread use, debate persists over unilateral vs. bilateral pedicle access. Both unilateral and bilateral approaches have their advantages and disadvantages. Bilateral access may allow symmetric cement distribution (5), whereas unilateral access can shorten operative time, reduce radiation exposure, and limit cement volume while still achieving cross-midline fill (i.e., cement spread crossing the vertebral midline, ensuring symmetric structural support of both vertebral halves) (6).

This study aimed to compare the clinical, radiographic, and procedural outcomes of unilateral vs. bilateral PVP performed under local anaesthesia in patients with single-level OVCF. To test these competing claims, we conducted a randomised trial under local anaesthesia comparing unilateral with bilateral PVP, assessing clinical and radiographic outcomes at baseline, discharge, and after 6 months. Both unilateral and bilateral PVP represent widely accepted standards of care for OVCFs. Consequently, randomisation was performed within a framework of clinical equipoise, ensuring that no participant was deprived of established treatment while allowing an unbiased comparison of two routinely used therapeutic strategies.

## 2 Materials and methods

### 2.1 Study design and participants

This study was designed as a prospective randomised controlled clinical trial comparing unilateral and bilateral PVP performed under local anaesthesia in patients with OVCFs. The study was conducted and reported in accordance with the CONSORT 2010 statement.

All eligible patients undergoing PVP between January 2022 and December 2024 were prospectively and consecutively enrolled in the study after confirmation of inclusion and exclusion criteria and after providing written informed consent. The inclusion and exclusion criteria are listed in Table 1.

**Table 1:** Inclusion and exclusion criteria for participants in the study.

Inclusion Criteria	Exclusion Criteria
Age ≥ 65 years at the time of diagnosis	Pathological vertebral fractures caused by metastatic disease, infection, or trauma
Single-level osteoporotic vertebral compression fracture (OVCF) confirmed by CT or MRI with corresponding localized back pain	Coagulopathy or use of anticoagulant therapy precluding safe puncture
Pain intensity ≥ 4 on the visual analogue scale (VAS) persisting despite ≥ 2 weeks of adequate conservative management (e.g., bed rest, bracing, or analgesics)	Secondary osteoporosis related to endocrine, metabolic, or inflammatory disorders
Ability and willingness to comply with scheduled follow-up assessments	Neurological deficit, radiculopathy, or spinal canal compromise on imaging
	Inability to tolerate local anaesthesia or maintain the prone position during surgery

The study protocol was approved by the Institutional Ethics Committee of the University Medical Centre Maribor (UKC-MB-KME-44/21) prior to patient enrolment and was conducted in accordance with the principles of the Declaration of Helsinki. The study was conducted as a prospective investigator-initiated clinical trial within routine clinical practice. Patient eligibility criteria, treatment allocation procedures, outcome measures, and the statistical analysis plan were predefined prior to study initiation and were not modified thereafter.

A total of 196 patients aged ≥65 years with single-level OVCFs were enrolled and randomised to a unilateral pedicular approach (*n* = 98) or a bilateral pedicular approach (*n* = 98). The mean age of the study population was 71.7 ± 6.5 years, with no significant difference between the unilateral and bilateral groups All vertebroplasty procedures were performed according to a standardized institutional protocol. In the unilateral group, PVP was performed through one pedicle on the more affected side, while in the bilateral group, PVP was performed through both pedicles. All procedures were performed under local anaesthesia with continuous fluoroscopic guidance. Patient positioning, anaesthesia technique, surgical approach, cement preparation, injection technique, fluoroscopic guidance, and postoperative management were applied consistently throughout the study period. The only procedural variable between groups was the pedicular access strategy (unilateral vs. bilateral). All procedures were performed by the same experienced spine surgeon to minimize inter-operator variability.

The trial was retrospectively registered at ClinicalTrials.gov (Identifier: NCT07198776; registered on 30 September 2025) to ensure transparency and public accessibility of the study protocol.

### 2.2 Randomisation and allocation concealment

Patients were prospectively randomized in a 1:1 ratio to unilateral or bilateral PVP using a computer-generated simple random allocation sequence. The randomisation list was generated prior to patient enrolment by an independent statistician who was not involved in patient recruitment, clinical care, surgical procedures, or outcome assessment. Allocation concealment was ensured using sequentially numbered, opaque, sealed envelopes prepared before patient enrolment. After confirmation of eligibility criteria and written informed consent, the next sequential envelope was opened in the operating theatre immediately prior to the procedure by a study nurse not involved in outcome evaluation. Due to the nature of the intervention, blinding of the operating surgeon and patients was not feasible. However, outcome assessors and data analysts were blinded to group allocation.

### 2.3 Surgical procedure

All procedures were performed in a dedicated operating theatre equipped with continuous biplanar fluoroscopic imaging (anteroposterior and lateral projections). The same experienced spine surgeon performed all interventions according to a standardized institutional protocol.

A 13-gauge vertebroplasty needle was used in all procedures. Under anteroposterior fluoroscopy, the entry point was identified at the lateral aspect of the pedicle, and the needle was advanced through the pedicle using alternating AP and lateral fluoroscopic control. The needle tip was positioned in the anterior third of the vertebral body prior to cement injection.

Polymethylmethacrylate (PMMA) cement (Confidence®, DePuy Synthes, Raynham, MA, USA) was prepared according to the manufacturer's instructions and injected during the doughy phase under continuous biplanar fluoroscopic monitoring. The average working time from mixing to injection was approximately 3–5 min.

#### 2.3.1 Patient positioning and local anaesthesia

Patients were placed in the prone position on a radiolucent table with chest and pelvic supports to allow abdominal decompression and reduce epidural venous pressure. Mild conscious sedation was administered when necessary while maintaining verbal communication throughout the procedure.

Local anaesthesia was achieved using stepwise infiltration of 1% lidocaine from skin to periosteum under fluoroscopic guidance. Attention was paid to adequate periosteal anaesthesia at the pedicle entry point to minimize discomfort during needle advancement.

#### 2.3.2 Unilateral transpedicular approach

**Figure 1:**
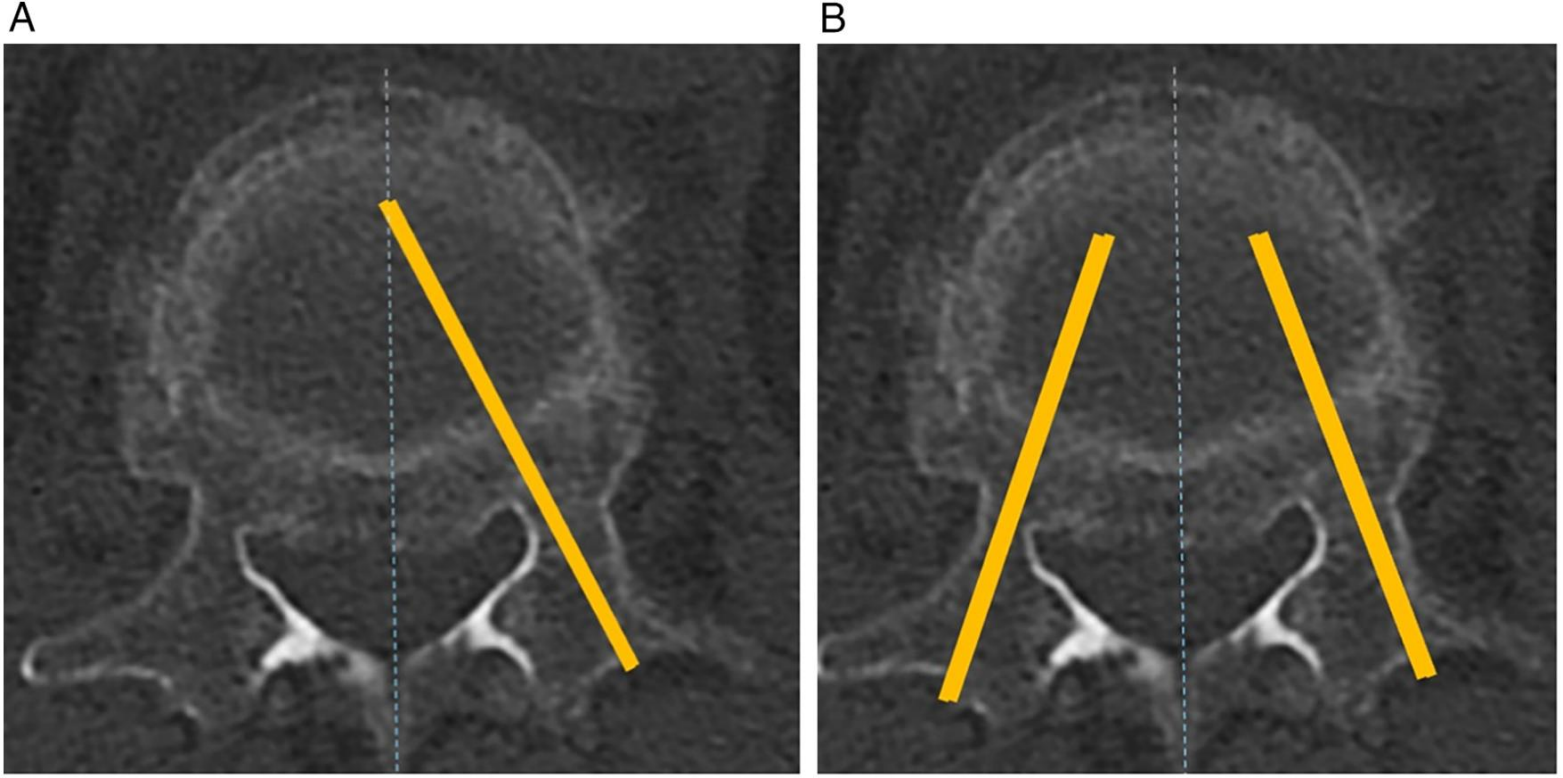
Schematic illustration of transpedicular needle trajectories during vertebroplasty.

In the unilateral group, a single transpedicular approach was performed on the more symptomatic or more collapsed side of the vertebral body. After identification of the pedicle entry point, the needle trajectory was directed medially under anteroposterior fluoroscopic guidance to reach or slightly cross the vertebral midline, facilitating cross-midline cement distribution (Figure 1A). Special emphasis was placed on optimizing the medial needle trajectory to achieve central cement dispersion through a single pedicle access. Care was taken to avoid excessive posterior needle positioning, thereby minimizing the risk of posterior cement leakage.

#### 2.3.3 Bilateral transpedicular approach

In the bilateral group, symmetric transpedicular access was established on both sides to achieve homogeneous cement distribution (Figure 1B). Each needle was advanced under fluoroscopic guidance to the anterior third of the vertebral body. Cement injection was performed alternately through both pedicles to achieve homogeneous bilateral filling. The medial needle trajectory on both sides was adjusted to ensure adequate coverage of the central vertebral body.

**(A)** Unilateral transpedicular approach demonstrating a more pronounced medial needle trajectory directed toward the vertebral midline to facilitate cross-midline cement distribution.
**(B)** Bilateral transpedicular approach showing symmetrical needle trajectories from both pedicles targeting the central vertebral body.

#### 2.3.4 Cement injection protocol

Injection was initiated during the doughy phase to reduce the risk of uncontrolled extravasation. Cement was injected slowly under continuous biplanar fluoroscopic monitoring. Injection was immediately discontinued if any of the following occurred:

- Cement approaching the posterior vertebral wall
- Venous extravasation
- Epidural extension
- Rapid cement migration

The procedural endpoint was adequate vertebral body filling with restoration of internal stability and, in unilateral cases, visible cross-midline cement spread on AP fluoroscopy.

#### 2.3.5 Criteria for adequate cross-midline fill

Cross-midline fill was defined as cement distribution extending beyond the vertebral midline on AP fluoroscopic view, ensuring structural support of both vertebral halves.

If satisfactory cross-midline distribution was not achieved through unilateral access despite optimal needle positioning, bilateral conversion would have been considered. However, in the present series, adequate cement dispersion was achieved in all unilateral cases without intraoperative conversion to bilateral access.

### 2.4 Clinical and radiographic assessment

Parameters analysed included patient demographics, operation time, radiation exposure time, amount of bone cement injection, cement leakage, pre- and post-procedural pain score assessed by visual analogue scale (VAS), Oswestry Disability Index (ODI), vertebral body height, kyphotic angle, and occurrence of adjacent level fractures. Baseline clinical and radiographic evaluation was performed preoperatively. Follow-up assessments were performed at baseline, at discharge (first postoperative day) and at 6 months postoperatively using identical standardized outcome measures in both study groups.

Cement leakage was evaluated intraoperatively using continuous biplanar fluoroscopic monitoring (anteroposterior and lateral views). Leakage patterns were classified according to the system described by Yeom et al. (7) based solely on fluoroscopic appearance. Cement extravasation was categorized into three types (Table 4):

- Type B (Basivertebral vein type): posterior leakage through the basivertebral venous system toward the spinal canal.
- Type S (Segmental vein type): leakage into the segmental venous system, typically extending laterally or paravertebrally.
- Type C (Cortical defect type): leakage through a cortical breach of the vertebral body, including anterior or lateral cortical defects.

In addition to Yeom classification, the anatomical location of cement extravasation was recorded (epidural, paravertebral, intradiscal, or neural foramen extension) based on fluoroscopic appearance. Only leakages clearly visible on fluoroscopy during the procedure were recorded. Minor venous streaks without progressive accumulation were not considered clinically relevant leakage. The presence of epidural or paravertebral extension was documented when clearly identifiable on fluoroscopic imaging. Postoperative CT imaging was not routinely performed in order to avoid additional radiation exposure, as fluoroscopic monitoring is considered standard practice for intraoperative detection of clinically relevant cement leakage.

### 2.5 Sample size calculation

Sample size calculation was performed using *G*Power 3.1* (University of Düsseldorf, Germany), based on detecting a minimum clinically significant difference of 1.5 VAS points between groups, assuming *α* = 0.05 and powe*r* = 0.8. The required sample size per group was 92 patients; therefore, 98 patients were recruited per arm to compensate for potential attrition and ensure adequate statistical power.

### 2.6 Statistical analysis

All randomised patients were analysed according to the intention-to-treat principle based on their initially assigned treatment group. Continuous variables were summarised as mean ± SD [or median (IQR) when non-normally distributed] and compared between groups using independent-samples t-tests or Mann–Whitney U tests; categorical variables were compared using Pearson's *χ*2 test (or Fisher's exact test when expected cell counts were < 5). Longitudinal changes in VAS (visual analogue scale) and ODI (Oswestry Disability Index) were analysed with repeated-measures ANOVA; sphericity was assessed with Mauchly's test, and Greenhouse–Geisser corrections were applied when violated. *post-hoc* pairwise comparisons were Bonferroni-corrected. Effect sizes were calculated using Cohen's d for continuous variables and Cramer's V for categorical variables to estimate the magnitude of between-group differences. Two-tailed *α* was set at 0.05; exact *p*-values are reported (software: IBM SPSS Statistics version 29.0 (IBM Corp., Armonk, NY, USA) and R version 4.3.2 (R Foundation for Statistical Computing, Vienna, Austria).

## 3 Results

### 3.1 Patient flow and baseline characteristics

**Figure 2:**
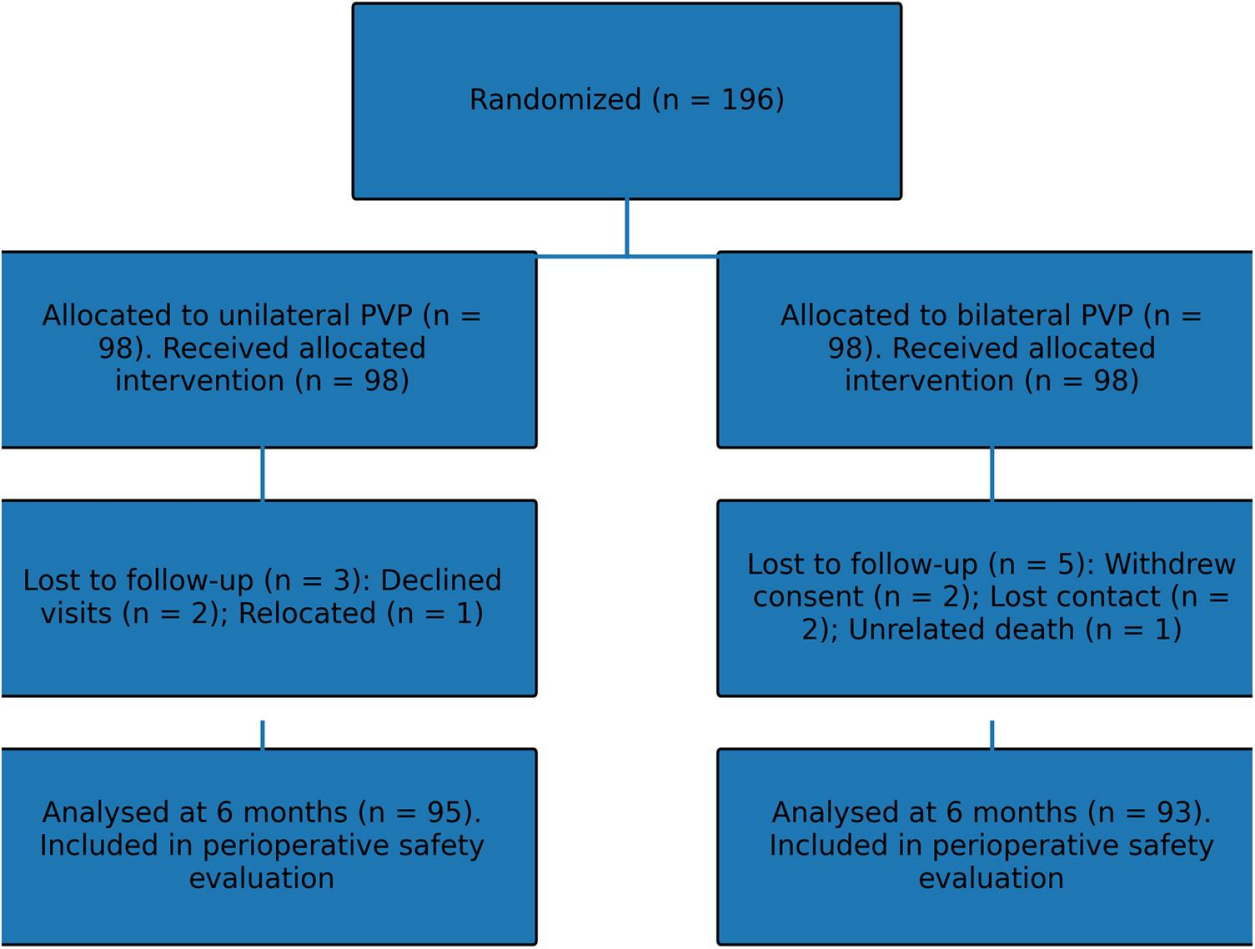
CONSORT flow diagram of patient enrolment, randomisation, follow-up and analysis.

Of the 196 randomised patients, 188 (95.9%) completed the 6-month follow-up. Eight patients were lost to follow-up: three in the unilateral group (two declined further visits, one relocated) and five in the bilateral group (two withdrew consent due to personal reasons, two were lost to contact, and one died of an unrelated cause). These patients were unavailable for the 6-month outcome analysis but were included in the perioperative safety evaluation. Patient enrolment, randomisation, follow-up, and analysis are summarised in Figure 2.

Baseline demographic and clinical characteristics were well balanced, with no statistically significant differences (Table 2). The mean age was 71.3 ± 6.0 years in the unilateral group and 72.1 ± 6.9 years in the bilateral group. Most patients were female (83.7% in unilateral PVP vs. 77.6% in bilateral PVP). Baseline pain and functional scores were comparable, with mean VAS 7.60 ± 0.47 vs. 7.66 ± 0.50 and ODI 60.4 ± 6.0 vs. 60.7 ± 5.6 in the unilateral and bilateral groups, respectively.

**Table 2:** Baseline demographic and clinical characteristics of patients.

Variable	Unilateral PVP (*n* = 98)	Bilateral PVP (*n* = 98)	*p*-value	Effect Size
Age (years), mean ± SD	71.3 ± 6.0	72.1 ± 6.9	0.378	Cohen's d = 0.13
Female sex, *n* (%)	82 (83.7%)	76 (77.6%)	0.281	Cramer's V = 0.08
Baseline VAS, mean ± SD	7.60 ± 0.47	7.66 ± 0.50	0.312	Cohen's d = 0.12
Baseline ODI, mean ± SD	60.4 ± 6.0	60.7 ± 5.6	0.694	Cohen's d = 0.05

PVP, Percutaneous Vertebroplasty; VAS, Visual Analogue Scale; ODI, Oswestry Disability Index.

### 3.2 Clinical outcomes

**Figure 3:**
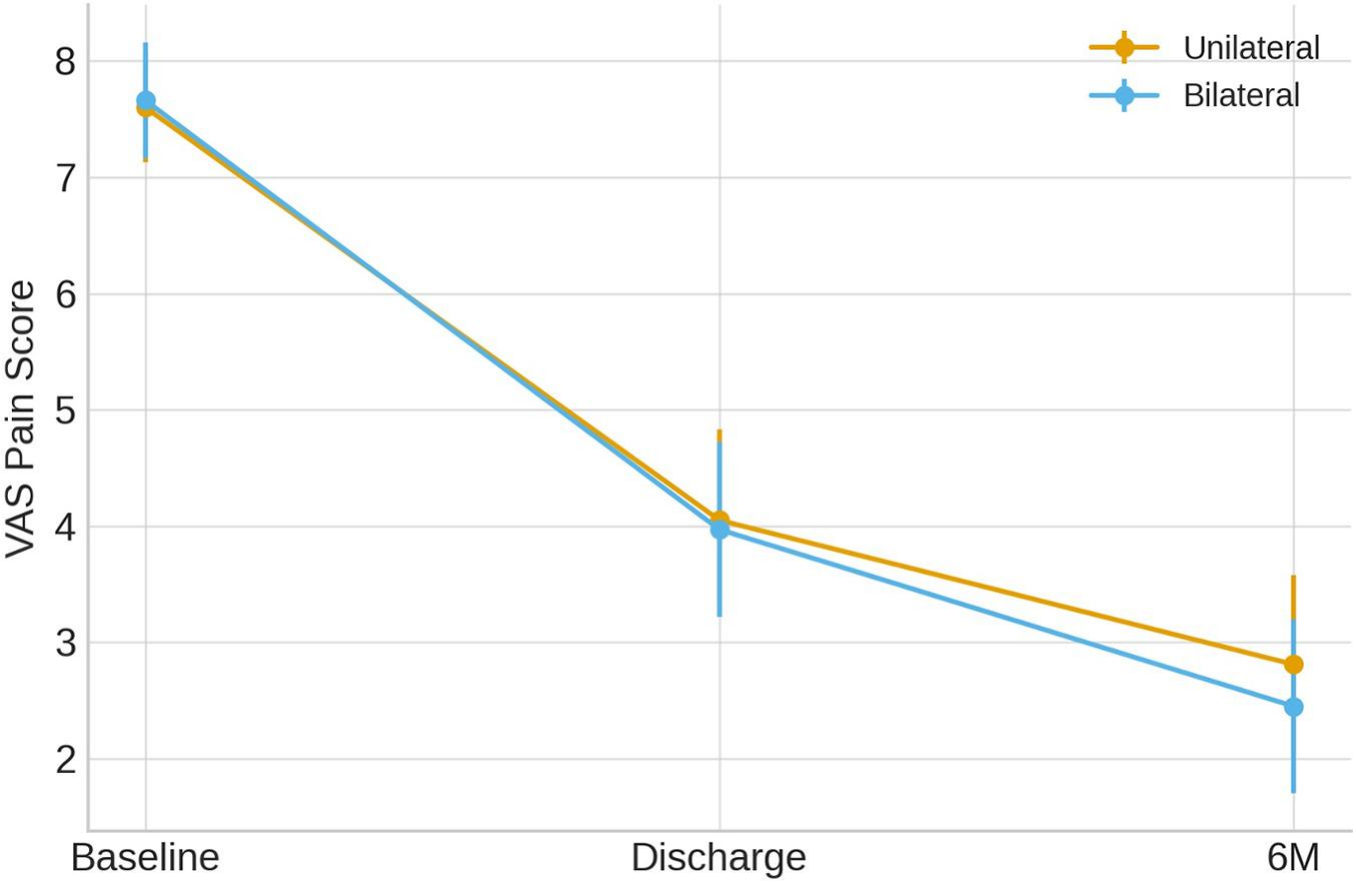
VAS scores over time in unilateral and bilateral PVP groups (mean ± SD). VAS, Visual Analogue Scale.

All patients underwent successful procedures under local anaesthesia without needing to stop the procedure due to pain and convert to general anaesthesia. Both techniques achieved rapid and significant pain relief, evident at discharge and sustained through the 6-month follow-up (Figure 3). In the unilateral group, mean VAS decreased from 7.60 ± 0.47 at baseline to 4.05 ± 0.78 at discharge and 2.81 ± 0.77 at 6 months, corresponding to a mean *Δ*VAS (baseline → 6 months) of 4.79 ± 0.86 points**.** In the bilateral group, VAS decreased from 7.66 ± 0.50 to 3.97 ± 0.75 at discharge and 2.45 ± 0.75 at 6 months, corresponding to a mean *Δ*VAS of 5.21 ± 0.88 points**.** The difference in *Δ*VAS between the two groups was not statistically significant (*p* = 0.084; Cohen's *d* = 0.24), indicating a comparable improvement in pain intensity over time.

**Figure 4:**
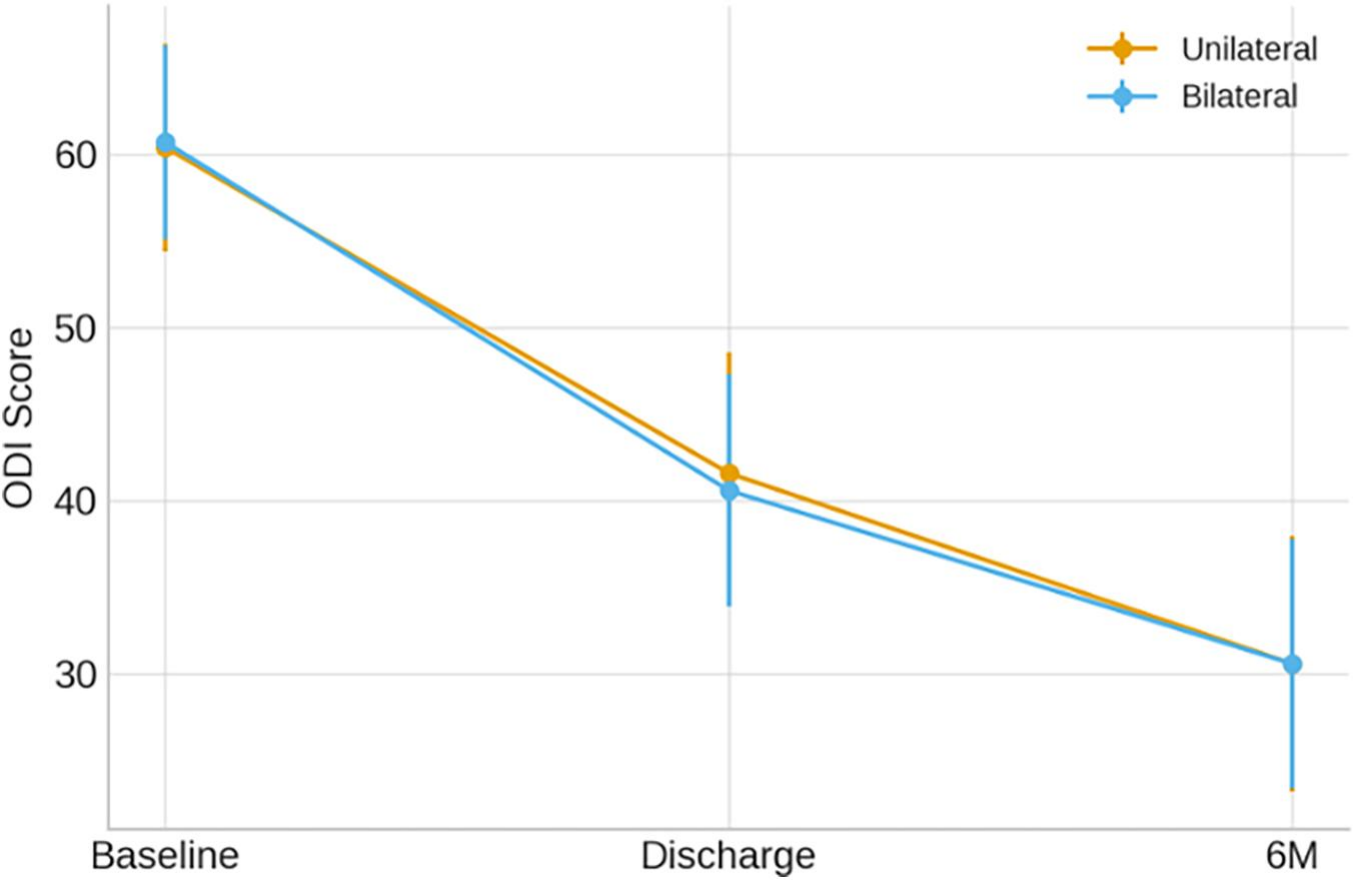
ODI scores over time in unilateral and bilateral PVP groups (mean ± SD).

Similarly, ODI demonstrated parallel improvement in both groups (Figure 4). Unilateral PVP patients improved from 60.4 ± 6.0 at baseline to 41.6 ± 7.0 at discharge and 30.6 ± 7.4 at 6 months. Bilateral PVP patients improved from 60.7 ± 5.6 to 40.6 ± 6.7 at discharge and 30.6 ± 7.2 at 6 months, corresponding to a mean *Δ*ODI of 30.1 ± 7.9 points**.** Again, the *Δ*ODI difference between groups was not statistically significant (*p* = 0.612; Cohen's *d* = 0.07), confirming similar functional recovery. At 6 months, all patients in both groups surpassed the minimal clinically significant difference (MCID) thresholds for pain (≥1.5 points in VAS) and disability (≥10 points in ODI).

### 3.3 Radiological outcomes

Radiological evaluations revealed no significant differences between the two approaches in terms of vertebral body height restoration or kyphotic angle correction (Table 3). The mean vertebral height restoration was 19.3 ± 3.3% in the unilateral PVP group compared with 18.9 ± 3.2% in the bilateral group (*p* = 0.418; Cohen's *d* = 0.12). Kyphotic angle correction averaged 5.9° ± 1.2° vs. 6.2° ± 1.1° (*p* = 0.293; Cohen's *d* = 0.26) in the unilateral and bilateral groups, respectively.

**Table 3:** Operative and radiological outcomes of unilateral versus bilateral PVP.

Variable	Unilateral PVP (*n* = 98) Mean ± SD	Bilateral PVP (*n* = 98) Mean ± SD	*p*-value	Effect Size
Operative time (min)	27.4 ± 2.4	42.4 ± 3.0	<0.001	Cohen's d = 1.22
Fluoroscopy time (s)	58.2 ± 9.0	93.8 ± 8.5	<0.001	Cohen's d = 1.10
Cement volume (mL)	3.9 ± 0.4	5.8 ± 0.5	<0.001	Cohen's d = 0.98
Vertebral height restoration (%)	19.3 ± 3.3	18.9 ± 3.2	0.418	Cohen's d = 0.12
Kyphotic angle correction (°)	5.9 ± 1.2	6.2 ± 1.1	0.293	Cohen's d = 0.26
Cement leakage, *n* (%)	7 (7.1%)	8 (8.2%)	0.742	Cramer's V = 0.03
Adjacent fractures, *n* (%)	5 (5.1%)	8 (8.2%)	0.401	Cramer's V = 0.07

PVP, percutaneous vertebroplasty; VAS, visual analogue scale; ODI, oswestry disability index; ns, not significant.

**Table 4:** Subclassification of cement leakage according to the yeom classification.

Leakage Type (Yeom)	Description	Unilateral PVP (*n* = 98)	Bilateral PVP (*n* = 98)	Clinical Consequences
Type B	Basivertebral venous leakage	4 (4.1%)	5 (5.1%)	None
Type S	Segmental venous leakage	2 (2.0%)	2 (2.0%)	None
Type C	Cortical defect leakage	1 (1.0%)	1 (1.0%)	None
Total leakage	All types	7 (7.1%)	8 (8.2%)	None

PVP, percutaneous vertebroplasty.

Leakage types classified according to Yeom et al. Percentages are calculated per treatment group.

### 3.4 Operative metrics

Operative metrics differed significantly between groups (Table 3). Operative time was markedly shorter with unilateral PVP (27.4 ± 2.4 min) compared to bilateral PVP (42.4 ± 3.0 min, *p* < 0.001; Cohen's d = 1.22). Fluoroscopy time was reduced by nearly 40% in the unilateral group (58.2 ± 9.0 s) relative to the bilateral group (93.8 ± 8.5 s, *p* < 0.001), corresponding to a *Δ* fluoroscopy time of 35.6 ± 12.5 s between groups. Cement volume was significantly lower with unilateral PVP (3.9 ± 0.4 mL) than with bilateral PVP (5.8 ± 0.5 mL, *p* < 0.001; Cohen's d = 0.98), representing a *Δ* cement volume of 1.9 ± 0.6 mL between groups.

Complication rates were low in both groups and showed no significant differences between the unilateral and bilateral PVP. Cement leakage was observed in 7.1% of unilateral and 8.2% of bilateral cases (*p* = 0.742; Cramer's V = 0.03), while new adjacent vertebral fractures within 6 months occurred in 5.1% and 8.2% of patients, respectively (*p* = 0.401; Cramer's V = 0.07). No cases of symptomatic pulmonary cement embolism, neurological injury, or other major adverse events were observed.

### 3.5 Clinical heterogeneity of single-level OVCFs

Although the present study included only single-level osteoporotic vertebral compression fractures, the treated population reflected the inherent clinical heterogeneity encountered in routine practice. Patients differed with respect to vertebral level involvement, degree of vertebral body collapse, fracture chronicity, and underlying osteoporosis severity, all of which may theoretically influence cement distribution and biomechanical stabilisation.

Despite this variability, the magnitude and trajectory of improvement in both pain (VAS) and functional disability (ODI) were consistent across the overall cohort. No clinical signal suggesting reduced efficacy of unilateral vertebroplasty in specific patient subsets was observed during follow-up. Both approaches resulted in sustained pain reduction, clinically meaningful functional recovery exceeding MCID thresholds, and comparable radiographic outcomes.These findings suggest that unilateral percutaneous vertebroplasty maintains clinical effectiveness across a heterogeneous spectrum of single-level OVCFs and supports its applicability in everyday clinical decision-making.

### 3.6 Procedural safety and complication profile

A detailed assessment of procedural safety was performed to further characterise complications beyond overall incidence rates. Perioperative and follow-up complications were systematically evaluated in both treatment groups.

Cement leakage occurred in 7 patients (7.1%) in the unilateral group and in 8 patients (8.2%) in the bilateral group (*p* = 0.742). According to the Yeom classification, in the unilateral group 4 cases were Type B (basivertebral venous), 2 were Type S (segmental venous), and 1 was Type C (cortical defect). In the bilateral group, 5 cases were Type B, 2 were Type S, and 1 was Type C. Most leakages represented venous extravasation without mass effect. No clinically significant epidural compression or symptomatic neural foraminal extension was observed. One Type C leakage in each group was limited to anterior cortical extravasation without posterior wall involvement. All leakage events were detected intraoperatively under continuous biplanar fluoroscopic monitoring. Cement injection was immediately halted upon identification of extravasation, and needle position was reassessed before considering continuation. In no case was decompression surgery, extended neurological observation, or additional imaging required.

Adjacent-level fractures occurred within the expected range reported for elderly osteoporotic populations and showed no statistically significant association with unilateral or bilateral access technique. Importantly, no difference in clinically relevant complication rates was observed between groups.

Overall, both unilateral and bilateral PVP demonstrated a favorable safety profile under local anaesthesia. These findings suggest that procedural safety is primarily influenced by patient-related factors, including bone quality and fracture biology, rather than pedicle access strategy, thereby supporting the feasibility of minimally invasive vertebral augmentation in frail elderly patients.

## 4 Discussion

The aim of this study was to compare the safety, procedural efficiency, and clinical efficacy of unilateral vs. bilateral percutaneous vertebroplasty (PVP) under local anaesthesia. The ideal treatment for OVCFs should provide rapid and sustained symptom relief, along with durable correction of the kyphotic deformity induced by the fracture (8). Considering that OVCFs are more common in vulnerable elderly patients with multiple pre-existing comorbidities (2), minimally invasive PVP performed under local anaesthesia represents an attractive treatment option, aiming to restore and maintain vertebral height and strength while rapidly alleviating pain and discomfort.

This randomised study demonstrated that both unilateral and bilateral PVP under local anaesthesia are safe and effective options for single-level OVCFs. Importantly, all patients tolerated local anaesthesia well, and none required conversion to general anaesthesia. This aligns with previous reports in the literature that PVP can be performed with the patient awake and with an acceptable comfort level (9). The ability to communicate with the patients also allows real-time monitoring of neural integrity by observing their responses (10). Taken together, the present results as well as data from the literature therefore support the use of a less invasive anaesthetic approach in elderly patients, where avoiding general anaesthesia reduces the risk of anaesthesia-related cardiac and pulmonary complications, postoperative delirium, and prolonged recovery room stays (11).

Traditionally, PVP has been performed by bilateral transpedicular approaches. More recently, the unilateral transpedicular approach has been gaining popularity (12). The superiority of bilateral vs. unilateral PVP has long been debated, mainly in relation to the adequacy of cement distribution and the potential for sustained vertebral body stabilisation. Concerns have been raised regarding unilateral access, especially with respect to achieving sufficient cross-midline cement fill and ensuring biomechanical stability (13). Nevertheless, biomechanical analyses suggest that when the needle trajectory is optimised, unilateral injection can achieve sufficient cement dispersion across the midline, providing stability comparable to bilateral injection (6). The present findings demonstrate that with careful optimization of medial needle trajectory and anterior vertebral body positioning, unilateral access reliably achieves cross-midline cement dispersion without the need for routine bilateral instrumentation.

In this study, both unilateral and bilateral PVP provided rapid and sustained improvements in pain and disability, as demonstrated by significant reductions in VAS and ODI scores from baseline to discharge and these improvements were maintained throughout the 6-month follow-up. The magnitude of improvement exceeded the accepted MCID thresholds for both VAS and ODI, highlighting that these were not only statistically significant but also clinically meaningful changes. The pattern of recovery was comparable between the two groups, with slightly lower mean VAS values at 6 months in the bilateral group. However, this difference did not reach statistical or clinical significance. ODI improvement was virtually identical between approaches, with both groups showing a reduction of nearly 50% compared with baseline values. These data therefore reinforce prior studies showing that PVP provides comparable efficacy of unilateral and bilateral approaches in terms of pain relief and functional recovery (14, 15). In line with this, a meta-analysis by Sun and Li (12) confirmed that unilateral and bilateral PVP achieve equivalent improvements in VAS and ODI, while unilateral access offers additional procedural efficiency. Taken together, these findings indicate that the clinical benefits of PVP in rapid pain relief and functional restoration are preserved regardless of unilateral or bilateral access.

Pain relief after PVP is believed to result from vertebral body stabilisation of micro movements and prevention of collapse. In this sense, it is reasonable to think that the more cement safely injected, the better results in terms of stability and pain relief. However, the true impact of cement volume on clinical outcomes remains controversial and is a subject of ongoing debate. In the present study, cement volume was significantly lower with unilateral PVP. These findings do not support the assumption that cement volume is the primary determinant of postoperative efficacy (16, 17). Additionally, no significant difference in cement leakage rates and adjacent vertebral fractures within 6 months were observed. These results, therefore, also do not support the assumption that larger volumes may increase the risk of leakage and potentially contribute to adjacent vertebral fractures (16).

To further characterize procedural safety, leakage events were subclassified according to the Yeom classification. Importantly, no posterior wall breach with epidural extension associated with neurological compromise was observed, supporting the procedural safety of both access strategies when performed under strict fluoroscopic control. The predominance of venous leakages (Type B and Type S) without mass effect reinforces the favorable safety profile of both approaches. These findings are consistent with previous literature indicating that the majority of cement extravasations during vertebral augmentation represent radiological findings without clinical relevance.

By approaching from both sides, cement fills the vertebra more evenly, achieving broader coverage and some biomechanical analyses, including a finite element study by Dai et al., suggest that bilateral cement augmentation better balances internal stresses and improves vertebral stability compared to unilateral, potentially reducing the risk of collapse under load (16, 18). This study, however, found no statistically significant differences between unilateral and bilateral groups in vertebral body height restoration or kyphotic angle correction. Some studies on percutaneous kyphoplasty have reported slightly greater kyphotic angle correction with bilateral approaches; however, these differences were minor and often clinically insignificant (19).

Importantly, in the present study, unilateral PVP demonstrated some procedural advantages, including reduced operative time and radiation exposure compared to the bilateral technique. The reduction in operative time by nearly 40% in the unilateral group has direct implications for operating room utilisation and patient safety, which is particularly relevant in elderly patients with multiple comorbidities. These findings are consistent with recent studies and meta-analyses (13, 20, 21), which confirmed the comparable efficacy and safety of unilateral and bilateral PVP under local anaesthesia and highlighted procedural efficiency advantages of the unilateral approach.

Radiation exposure is another important consideration. Unilateral PVP consistently reduces fluoroscopy time and cumulative radiation dose, as confirmed in this trial and prior reports (22). Given the high prevalence of OVCFs and the frequency of vertebral augmentation procedures, reducing radiation exposure benefits both patients and surgical staff and contributes to long-term occupational safety.

From a practical clinical perspective, the choice between unilateral and bilateral PVP is rarely determined by fracture classification alone. Instead, intraoperative factors such as cement flow pattern, achievement of cross-midline cement distribution, pedicle anatomy, and real-time fluoroscopic assessment play a decisive role. The present finding suggests that unilateral PVP can be considered an effective first-line strategy in most single-level OVCFs, while bilateral access may remain appropriate in selected cases where satisfactory cement dispersion cannot be achieved through a unilateral trajectory.

Subsequent adjacent-level fractures in 6-month follow-up occurred within expected range reported for elderly osteoporotic populations and showed no relationship to the unilateral or bilateral access technique. This observation is in line with prior randomised studies (23), suggesting that the risk of adjacent fractures is more strongly influenced by baseline osteoporosis and biomechanical factors rather than pedicle access strategy, and does not support the assumption that larger cement volumes may increase the risk of leakage and potentially contribute to adjacent vertebral fractures.

Based on the findings of the present randomised study and existing literature (12, 20, 21) a pragmatic decision approach may be proposed. Unilateral PVP may be preferred as the initial strategy in single-level OVCFs due to reduced operative time, lower radiation exposure, and comparable clinical outcomes. Bilateral access may be considered when unilateral needle positioning fails to achieve adequate midline cement distribution, in cases of severe vertebral collapse, complex pedicle anatomy, or insufficient intraoperative stabilisation. Such an individual approach emphasizes procedural adaptability rather than routine bilateral instrumentation.

Despite its strengths, including a prospective randomised design and uniform local anaesthesia protocol, the present study has several limitations. First, the follow-up period was limited to six months. Although this timeframe is sufficient to assess short-term clinical outcomes and procedural safety, longer follow-up is required to evaluate the durability of pain relief, radiographic stability, and incidence of new vertebral fractures. Second, this study was conducted at a single centre, which may limit generalizability across different patient populations and surgical teams. Third, while fluoroscopy time was used as a proxy for radiation exposure, direct radiation dose measurements (to both patient and staff) would provide more precise safety data. Finally, this study did not perform subgroup analyses stratified by fracture morphology, degree of vertebral collapse, or spinal deformities such as scoliosis, which may influence cement distribution and clinical outcomes. In addition, the trial was retrospectively registered; however, the study protocol and statistical analysis plan were predefined prior to study initiation and remained unchanged throughout the study. Finally, all procedures were performed by a single experienced spine surgeon, which may limit generalizability to other clinical settings but ensured procedural consistency and minimized technical variability between treatment groups.

## 5 Conclusions

This randomised trial demonstrates that both unilateral and bilateral PVP performed under local anaesthesia provide comparable clinical and radiographic outcomes in patients with single-level OVCFs. Unilateral PVP conferred several procedural advantages, including shorter operative time, reduced cement volume, and lower radiation exposure, without compromising efficacy or safety at short-term follow-up. Complication rates, including cement leakage and adjacent-level fractures, were low in both groups and showed no significant differences. These findings suggest that unilateral PVP under local anaesthesia may serve as an efficient first-line approach for most single-level OVCFs, offering equivalent clinical benefits while improving procedural efficiency and resource utilisation, while bilateral access remains a valuable option when optimal cement distribution cannot be achieved through a unilateral trajectory.

Future research should focus on large multi-centre randomised controlled trials with extended follow-up durations of at least 12–24 months, to confirm the long-term equivalence of outcomes between approaches. Further biomechanical studies and advanced imaging analyses could clarify whether specific fracture morphologies necessitate bilateral access to ensure optimal cement distribution and may further refine patient selection criteria

## Data Availability

The original contributions presented in the study are included in the article/Supplementary Material, further inquiries can be directed to the corresponding author.
